# Draft Genome Sequence of *Rhodococcus erythropolis* NSX2, an Actinobacterium Isolated from a Cadmium-Contaminated Environment

**DOI:** 10.1128/genomeA.01147-16

**Published:** 2016-10-20

**Authors:** Eleonora Egidi, Jennifer L. Wood, Edward M. Fox, Wuxing Liu, Ashley E. Franks

**Affiliations:** a Department of Physiology, Anatomy and Microbiology, La Trobe University, Bundoora, Victoria, Australia; b CSIRO Agriculture and Food, Werribee, Victoria, Australia; c Key Laboratory of Soil Environment and Pollution Remediation, Institute of Soil Science, Chinese Academy of Sciences, Nanjing, China

## Abstract

*Rhodococcus erythropolis* NSX2 is a rhizobacterium isolated from a heavy metal–contaminated environment. The 6.2-Mb annotated genome sequence shows that this strain harbors genes associated with heavy-metal resistance and xenobiotics degradation.

## GENOME ANNOUNCEMENT

*Actinobacteria* represent a dominant fraction of the metabolically active rhizosphere population in several heavy-metal (HM) accumulators (1). Members of this phylum are able to produce both metal-mobilizing and metal-immobilizing compounds (2), a crucial requirement to increase the HM extractability (3). This suggests an important role of *Actinobacteria* in metal uptake and translocation during phytoextraction (4). Nevertheless, our knowledge on the mechanisms of HM solubilization and resistance in the phylum is still limited. In this paper, we report the draft genome sequence of *Rhodococcus erythropolis* NSX2, a Gram-positive actinobacterium isolated from the rhizosphere of the heavy-metal hyperaccumulator *Sedum X Graptosedum* grown in a cadmium-contaminated environment (5). NSX2 was shown to be resistant to a MIC of 600 mg·L^−1^ for Cd (5). The draft genome sequence will shed new light on the mechanism underlying the heavy-metal resistance, plant-growth promotion, and degradation abilities of *R. erythropolis* NSX2.

The strain was grown in cell culture, and the total genomic DNA was extracted from purified *R. erythropolis* NSX2 and converted to sequencing libraries using the Nextera XT DNA library preparation kit (Illumina). Libraries were normalized and pooled before sequencing on an Illumina MiSeq with 2 × 300-bp paired-end reads. For each isolate, the A5-miseq pipeline (6) was used to perform read trimming and correction, contig assembly, crude scaffolding, misassembly correction, and final scaffolding. The total length of the assembled genome was 6,279,737 bp, with a G+C content of 62.4% and median coverage of 66×. The final number of contigs was 37 with an *N*_50_ value of 608,915.

A total of 5,963 candidate protein-coding genes were identified by automated annotation of the *R. erythropolis* NSX2 draft genome sequence using RAST (7). Comparative genome analysis revealed that *R. erythropolis* PR4 234621.6 (GenBank accession no. GCA_000010105.1) is NSX2’s closest neighbor (score = 532). The NSX2 genome retains several traits associated with the uptake, efflux, reduction, and oxidation of metal ions. These include genes coding for the cobalt-zinc-cadmium resistance proteins CzcA and CzcD, the cation efflux system protein CusA, an arsenate reductase, an arsenic efflux pump protein, the copper resistance protein CopC, and a transcriptional regulator for the mercury resistance protein MerR*.*

Interestingly, the NSX2 genome also harbors genes associated with the metabolism of aromatic compounds. In particular, we identified predicted genes encoding for proteins involved in the catabolism of protocatechuate and catechol (e.g., beta-ketoadipyl CoA thiolase, potocatechuate 3,4-dioxygenase beta chain, and beta-ketoadipate enol-lactone hydrolase), as well as proteins linked to the biphenyl degradation process (e.g., biphenyl-2,3-diol 1,2-dioxygenase, and 2-hydroxy-6-oxo-6-phenylhexa-2,4-dienoate hydrolase). The presence of these genetic markers suggests the ability of *R. erythropolis* NSX2 to metabolize many plant-derived compounds, as well as potentially degrade environmental pollutants (8).

### Accession number(s).

This whole-genome shotgun project has been deposited at DDBJ/ENA/GenBank under the accession number MDCH00000000. The version described in this paper is the first version, MDCH01000000.
